# Beyond social media time: online self-disclosure, depressive symptoms, somatic complaints, and suicidal ideation in adolescents

**DOI:** 10.3389/fpsyt.2026.1926710

**Published:** 2026-09-23

**Authors:** Grazia Maria Giovanna Pastorino, Antonio Aquino, Rossella Suriano, Roberto Buonaiuto, Giuseppe Diaspro, Miriam Olivieri, Noemi Mungo, Francesca Felicia Operto

**Affiliations:** 1 Department of Health Sciences, Magna Graecia University of Catanzaro, Catanzaro, Italy; 2 Clinical and Translational Oncology, Scuola Superiore Meridionale, Naples, Italy; 3 Department of Breast & Thoracic Oncology, Division of Breast Medical Oncology, Istituto Nazionale Tumori Istituto di Ricovero e Cura a Carattere Scientifico (IRCSS) ‘Fondazione G. Pascale’, Naples, Italy; 4 Department of Clinical Medicine and Surgery, University of Naples Federico II, Naples, Italy; 5 Department of Mental Health, Physical Health and Preventive Medicine, University of Campania “L.Vanvitelli”, Naples, Italy; 6 Salerno Medical School, University of Salerno, Baronissi, Italy

**Keywords:** adolescents’, depressive symptoms, private online self-disclosure, social media, somatization, suicidal ideation

## Abstract

**Introduction:**

Screen time alone may not adequately capture adolescents’ digital vulnerability. This study examined whether private online self-disclosure, depressive symptoms, and somatic complaints were associated with suicidal ideation beyond time spent on social media.

**Methods:**

A school-based cross-sectional study was conducted in 1364 adolescents aged 11–19 years. Participants completed a questionnaire on social media use and private online self-disclosure, the Children’s Depression Inventory–2, and selected Youth Self-Report items assessing somatic complaints and suicidal ideation. Correlation and hierarchical regression analyses were performed.

**Results:**

Suicidal ideation was associated with depressive symptoms, somatic complaints, and private online self-disclosure. In the final regression model, depressive symptoms, somatic complaints, and private online self-disclosure remained independently associated with suicidal ideation, whereas time spent on social media was not significantly associated with suicidal ideation after adjustment for the other predictors. Somatic complaints explained additional variance beyond depressive symptoms and social media-related variables.

**Conclusions:**

Private online self-disclosure and somatic complaints were associated with greater psychological vulnerability beyond time spent on social media, although their predictive and clinical utility requires further investigation in prospective studies.

## 1 Introduction

Adolescent mental health disorders have markedly increased over the past decade and currently represent a major global public health concern. Approximately one in seven individuals aged 10–19 years experiences a mental disorder, with anxiety and depressive conditions ranking among the leading causes of disability in this age group (1, 2). Suicide is now one of the primary causes of death among adolescents and young adults worldwide (2). In pediatric clinical practice, this epidemiological trend translates into a growing number of consultations for emotional distress, behavioral dysregulation, and medically unexplained physical symptoms that often conceal underlying psychological vulnerability. Because early psychopathological manifestations frequently persist into adulthood, the identification of modifiable psychosocial risk factors has become a priority for preventive pediatric care.

One of the most influential environmental factors shaping contemporary adolescent development is the digital ecosystem. Social media platforms are deeply embedded in youths’ daily lives, with the majority of adolescents reporting daily use (3). Although these platforms facilitate social connection and identity exploration, they also expose adolescents to several psychosocial risks. A substantial proportion of youths exhibit patterns consistent with Problematic Social Media Use (PSMU), characterized by impaired control, withdrawal symptoms, and functional impairment (3). During adolescence, a developmental stage marked by heightened sensitivity to peer evaluation and identity formation, digital environments become central arenas for social comparison and self-presentation (4).

Extensive literature has documented associations between intensive social media engagement and internalizing symptoms, particularly depression (5–7). However, the relationship between digital behaviors and suicidality remains less clearly defined, suggesting the presence of complex mediating mechanisms (8). This association is likely bidirectional: excessive social media exposure may exacerbate depressive symptoms through cyberbullying, social comparison, and emotional overstimulation, while psychologically vulnerable adolescents may increasingly rely on digital environments as maladaptive coping strategies (9).

Beyond psychological outcomes, adolescent psychological distress may also be expressed through somatic complaints. Headaches, fatigue, nausea, dizziness, and recurrent pain symptoms are common in pediatric and school-based settings and may reflect underlying emotional vulnerability, particularly when no clear medical explanation is identified (10). In this context, somatic complaints may represent a clinically relevant manifestation of internalizing distress and a potential indicator of psychological vulnerability. This is particularly important because pediatricians are often among the first healthcare professionals to evaluate adolescents presenting with medically unexplained physical symptoms, placing them in a strategic position for early identification of emotional distress and suicide-related vulnerability.

Recent research further suggests that the qualitative characteristics of digital engagement may be more clinically informative than mere exposure time. Rather than focusing exclusively on how much time adolescents spend online, increasing attention has been given to the psychological meaning and function of online behaviors. In particular, adolescents may use digital environments to express private aspects of themselves, seek emotional validation, or disclose experiences that are difficult to share in face-to-face relationships. Although online self-expression is not inherently maladaptive, a marked discrepancy between online and offline disclosure may indicate unmet relational needs, social withdrawal, fear of judgment, or reduced access to trusted offline support (11, 12).

Despite growing societal concern about minors’ social media use, clinical practice requires a more nuanced understanding of the mechanisms linking specific digital behaviors to severe mental health outcomes. Approaches based exclusively on reducing screen time may overlook adolescents whose vulnerability is reflected not in the amount of time spent online, but in the psychological function of their online engagement. Clarifying these associations is essential for developing clinically meaningful screening strategies and preventive interventions in pediatric and school-based settings.

Theoretical framework.

The present study was grounded in an integrated developmental and clinical framework linking digital engagement, emotional distress, somatic complaints, and suicidal ideation in adolescence. First, the Differential Susceptibility to Media Effects Model suggests that media effects are not uniform, but depend on dispositional, developmental, and social susceptibility factors (13). From this perspective, the psychological meaning of social media use may be more relevant than exposure time alone. Adolescents with greater emotional vulnerability or reduced offline support may therefore be more susceptible to maladaptive digital experiences, whereas similar levels of screen time may have different implications across individuals.

Second, the Compensatory Internet Use Model provides a useful framework for interpreting private online self-disclosure (14). According to this model, individuals may use online environments to compensate for unmet psychological or relational needs, to regulate negative affect, or to manage difficulties experienced in offline contexts. In adolescence, when identity formation, peer acceptance, and emotional validation are central developmental tasks, online spaces may offer a controllable setting for self-expression and experimentation (15). Thus, disclosing private experiences online that are not shared offline should not be considered inherently maladaptive. However, when online disclosure becomes a privileged or exclusive channel for expressing personal distress, it may reflect social withdrawal, fear of judgment, shame, or limited access to trusted offline relationships.

Third, theories of adolescent identity development in the digital age emphasize that digital environments are not merely contexts of exposure, but spaces in which adolescents explore identity, seek feedback, and negotiate belonging (15). Online self-disclosure may therefore represent an attempt to achieve authenticity, connection, or emotional validation. At the same time, a marked discrepancy between online and offline self-expression may indicate difficulties integrating personal experiences within everyday relationships. This discrepancy may be clinically relevant when it co-occurs with depressive symptoms or somatic complaints, suggesting that psychological distress remains insufficiently recognized or verbalized in offline contexts.

Finally, suicide theories provide a clinical rationale for linking private online self-disclosure, depressive symptoms, somatic complaints, and suicidal ideation. The Interpersonal Theory of Suicide emphasizes the role of thwarted belongingness and perceived burdensomeness in the emergence of suicidal desire (16), whereas the Integrated Motivational–Volitional Model highlights how feelings of defeat, entrapment, and poor social connectedness may contribute to suicidal ideation (17). Within this framework, depressive symptoms may represent a core affective dimension of suicidal vulnerability, while somatic complaints may reflect an embodied expression of psychological distress. Private online self-disclosure may therefore reflect unmet emotional or relational needs rather than acting as a direct causal risk factor. Taken together, these models support the hypothesis that qualitative aspects of digital engagement, depressive symptoms, and somatic complaints may identify adolescents with greater suicidal vulnerability beyond the amount of time spent on social media.

The present study investigated the associations between social media use patterns, private online self-disclosure, depressive symptoms, somatic complaints, and suicidal ideation in a large school-based adolescent population. Given the cross-sectional design, the study did not aim to establish causal or temporal pathways. Rather, it aimed to examine whether qualitative aspects of digital engagement, particularly the tendency to disclose private experiences online that are not shared offline, were associated with suicidal ideation beyond time spent on social media. A further aim was to evaluate whether somatic complaints accounted for additional variance in suicidal ideation after considering depressive symptoms and social media-related variables, thereby examining the potential clinical relevance of somatic complaints in adolescents’ psychological vulnerability.

Based on this framework, four hypotheses were formulated.

H1: Higher levels of private online self-disclosure, depressive symptoms, and somatic complaints would be associated with higher levels of suicidal ideation in adolescents.
H2: Private online self-disclosure would show a more consistent association with suicidal ideation than time spent on social media, supporting the relevance of qualitative aspects of digital engagement beyond screen time.
H3: Depressive symptoms would explain additional variance in suicidal ideation beyond demographic characteristics, time spent on social media, and private online self-disclosure.
H4: Somatic complaints would explain additional variance in suicidal ideation beyond depressive symptoms, time spent on social media, and online self-disclosure, suggesting that somatic complaints may provide incremental information about adolescents’ psychological vulnerability.

## 2 Methods

### 2.1 Ethics approval and consent to participate

The study was approved by the Campania Sud Ethics Committee (protocol number = 0033986). All procedures were conducted in accordance with the ethical standards of the responsible institutional committee and with the 1964 Helsinki Declaration and its later amendments.

For participants aged under 18 years, written informed consent was obtained from parents or legal guardians prior to data collection, and written assent was obtained from the adolescents. Participants aged 18 and 19 years provided independent written informed consent.

Given the sensitive nature of items assessing depressive symptoms and suicidal thoughts, data were collected anonymously to ensure response authenticity and reduce social desirability bias. To safeguard participant well-being, dedicated informative sessions were conducted with the students prior to questionnaire administration. During these meetings, researchers provided contact details for national crisis helplines (e.g., Telefono Azzurro, national emergency lines) and local mental health services. Furthermore, students were explicitly given the opportunity to contact the project leaders—a multidisciplinary team of specialists in Child and Adolescent Psychiatry and clinical psychologists—for immediate, confidential support or referral if they experienced psychological distress.

### 2.2 Study design and sample selection

A school-based cross-sectional study was conducted across six middle and high schools in the Campania region of Southern Italy. A total of 1, 436 adolescents were initially invited to participate. Of these, 72 were excluded (30 due to lack of parental consent or student refusal, and 42 due to invalid or substantially incomplete survey responses). The final recruited sample comprised 1, 364 adolescents aged 11–19 years (653 males, 48%; 696 females, 51%; 15 undeclared, 1%; mean age = 14.99 ± 2.15 years). Questionnaires were completed via paper format by 1, 000 participants and via Google Forms by 364 participants. Complete data for the primary regression model were available for 1, 160 adolescents.

Participants aged 18 and 19 years (n = 194, 14.2% of the total sample) were high school students enrolled in final-year classrooms during school-based cluster sampling. To ensure that including students beyond the standardized normative age boundaries of the tools (CDI-2: 7–17 years; YSR: 11–18 years) did not introduce normative scoring bias, a sensitivity analysis restricting the sample strictly to adolescents aged 11–17 years was conducted.

Participation was voluntary. Adolescents were eligible if they were able to understand and complete the questionnaires. Participants with severe medical or neuropsychiatric conditions that could compromise questionnaire comprehension, despite support, were excluded. The sample was characterized by widespread social media use: smartphones were the most commonly used device for Internet access (90%), and the most frequently reported reasons for Internet use were social networking (72%), listening to music (51%), school-related activities (30%), and video gaming (24%). Instagram was the most commonly used platform (82%), followed by YouTube (56%) and Facebook (15%).

Parents or legal guardians were informed about the aims and procedures of the study and provided written informed consent. Preliminary meetings were also held with school principals and teachers to explain the study procedures. Data were collected anonymously using either paper questionnaires or Google Forms.

### 2.3 Measurement

Participants completed a study-specific questionnaire assessing demographic characteristics and social media use, the Children’s Depression Inventory–Second Edition Self-Report form (CDI-2 SR) (18), and selected items from the Achenbach System of Empirically Based Assessment Youth Self-Report/11–18 (YSR/11–18) assessing somatic complaints and suicidal ideation (19).

#### 2.3.1 Social media use and private online self-disclosure

Social media use was assessed using a study-specific questionnaire developed by the research team to explore quantitative and qualitative aspects of adolescents’ digital engagement.

Time spent on social media was assessed with the item: “How much time do you spend on social media on average each day?” Responses were coded on a five-level ordinal scale: 0 = never, 1 = less than 1 hour per day, 2 = 1–3 hours per day, 3 = 4 hours or more per day (reflecting heavy active daily use), and 4 = always connected (reflecting continuous, immersive engagement and constant background presence throughout the day). Higher scores indicated greater daily social media exposure.

Private online self-disclosure was assessed using three items focused on adolescents’ tendency to disclose or express personal experiences online that are not shared offline: “Do you talk about private things on the Internet that you would not share with others in person?”; “Do you think it is easier to be yourself on the Internet than in person?”; and “Can you talk about more things online than you do in person?”. Each item was coded on a three-level scale: 0 = never, 1 = sometimes, and 2 = often.

A composite private online self-disclosure score was computed by summing the three items, with a theoretical range from 0 to 6. Higher scores indicated a greater tendency to disclose private or personal experiences online that are not shared offline. The three items assess behavioral disclosure, perceived online authenticity, and breadth of digital communication. Internal consistency for this 3-item study-specific scale was evaluated, yielding a Cronbach’s alpha of.57 and an average inter-item correlation of r = .31 (p <.001), which is acceptable for a very brief screening indicator. Because this questionnaire was developed for the present study, these variables were interpreted as study-specific indicators of digital engagement rather than as standardized diagnostic measures.

#### 2.3.2 Depressive symptoms

Depressive symptoms were assessed using the Children’s Depression Inventory–Second Edition Self-Report form (CDI-2 SR) (18). The CDI-2 is a standardized self-report questionnaire designed to assess depressive symptoms in children and adolescents. It includes 28 items rated on a Likert-type scale from 0 to 2, with higher scores indicating greater depressive symptom severity.

The CDI-2 provides a Total Score and subscale scores assessing emotional and functional problems, including Emotional Problems, Negative Mood/Physical Symptoms, Negative Self-Esteem, Functional Problems, Ineffectiveness, and Interpersonal Problems. Raw scores can be converted into T-scores according to normative data. In the present study, the CDI-2 Total Score was used as a continuous measure of depressive symptoms.

In addition to the standard CDI-2 Total Score, a refined depressive symptom score was calculated by subtracting Item 8 (suicidality item: ‘I do not think about killing myself’/’I think about killing myself but I would not do it’/’’I want to kill myself’) to evaluate model stability and eliminate potential item overlap with the dependent variable.

#### 2.3.3 Somatic complaints and suicidal ideation

Somatic complaints and suicidal ideation were assessed using selected items from the Achenbach System of Empirically Based Assessment Youth Self-Report/11–18 (YSR/11–18) (19). The YSR/11–18 is a standardized self-report questionnaire designed to assess emotional and behavioral problems in adolescents. Items are rated on a three-point scale: 0 = not true, 1 = somewhat or sometimes true, and 2 = very true or often true.

Somatic complaints were assessed by summing five selected YSR items referring to physical symptoms without clear medical cause: dizziness, headache, nausea or feeling sick, eye problems, and vomiting or retching. The resulting somatic complaints score ranged from 0 to 10, with higher scores indicating a greater burden of physical symptoms (Cronbach’s alpha of.66).

Suicidal ideation was assessed using the YSR item “I think about killing myself, “ rated on the same 0–2 scale. This item was used as the dependent variable in the main analyses, with higher scores indicating greater suicidal ideation.

### 2.4 Statistical analysis

Statistical analyses were performed using IBM SPSS Statistics for Windows, version 28.0 (IBM Corp., Armonk, NY, USA). Descriptive statistics were used to summarize demographic characteristics, social media use patterns, depressive symptoms, somatic complaints, and suicidal ideation. Continuous variables were reported as means and standard deviations, whereas categorical and ordinal variables were reported as frequencies and percentages.

Bivariate associations among suicidal ideation, somatic complaints, depressive symptoms, time spent on social media, and private online self-disclosure were examined using Pearson correlation analyses.

To examine the incremental association of digital engagement variables, depressive symptoms, and somatic complaints with suicidal ideation, a hierarchical linear regression analysis was conducted.

Suicidal ideation was treated as a dimensional score ranging from 0 to 2. Predictors were entered in theoretically defined blocks rather than as a temporal sequence. In Model 1, age and gender were entered as demographic covariates. Gender was coded as a binary variable, with males coded as 0 and females coded as 1; participants with undeclared gender were treated as missing in analyses including gender. In Model 2, time spent on social media and private online self-disclosure were added. In Model 3, depressive symptoms, measured using the CDI-2 Total Score, were entered. In Model 4, somatic complaints was added to test whether somatic symptoms explained additional variance in suicidal ideation beyond demographic characteristics, social media-related variables, and depressive symptoms.

For each regression model, unstandardized coefficients, standardized beta coefficients, standard errors, t values, p values, R², adjusted R², and changes in explained variance were reported. Multicollinearity was evaluated using tolerance and variance inflation factor values. Residual normality and homoscedasticity were visually inspected using P-P plots and scatterplots of standardized residuals against predicted values. To ensure that item overlap between the CDI-2 suicidality item and the dependent variable did not artifactually inflate predictor relationships, all multivariable models were re-estimated as a sensitivity analysis using the refined CDI-2 score (excluding Item 8).

To confirm model stability across the standardized normative age ranges of the psychometric tools, a secondary sensitivity analysis was executed restricting the hierarchical regression model to participants aged 11–17 years (N = 978).

Missing data for individual survey items was low (0% for CDI-2 score and somatic complaints, 0.95% for social media time, 1.1% for gender, 2.35% for online self-disclosure, 3.81% for suicidal ideation, and 8.14% for participant age). Listwise deletion across all seven variables yielded a final analytic sample of 1, 160 adolescents (85% of the total recruited sample). To evaluate potential attrition bias, independent-samples t-tests and Chi-square tests were conducted comparing included (n = 1, 160) and excluded (n = 204) participants. No statistically significant differences were observed between included and excluded participants regarding age (p = .105), gender distribution (p = .217), depressive symptoms (p = .240), suicidal ideation (p = .244), or private online self-disclosure (p = .447), confirming that listwise deletion did not introduce systematic bias on primary clinical outcomes.

To account for the three-level ordinal nature of the suicidal ideation outcome (0 = not true, 1 = somewhat/sometimes true, 2 = very/often true) and evaluate the robustness of the primary linear regression model, a secondary sensitivity analysis using ordinal logistic regression was conducted. The proportional odds assumption was evaluated using the test of parallel lines. Unstandardized regression coefficients (B), standard errors (SE), z-values, p-values, Odds Ratios (OR), and 95% Confidence Intervals (95% CI) were reported.

Results were considered statistically significant at p <.05.

## 3 Results

### 3.1 Sample characteristics

Subject enrollment was voluntary, and the participation rate in the participating schools was 95%. The questionnaires were administered in a paper format for 1000; instead, 364 questionnaires were filled out on a multimedia platform via Google forms. The main sample characteristics are summarized in **Table 1**.

**Table 1:** Sample characteristics.

Characteristic	n (%) / Mean ± SD
Sample Size - n	1364
Gender	
male - n (%)	653 (48%)
female n (%)	696 (51%)
undeclared - n (%)	15 (1%)
Age years	14.99 ± 2.15

SD, Standard Deviation.

m, mean; SD, standard deviation.

### 3.2 Descriptive statistics of social media questionnaire

All the results that describe the modality of Social Media use by adolescents, explored through the Social Media Questionnaire, were summarized in **Table 2**.

**Table 2:** Social media questionnaire.

Question	Response
How much time do you spend on Social Media on average each day?	Never =34 (2.5%) Less than 1 hour = 193 (14%) 1 to 3 hours = 603 (44%) 4 hours or more = 275 (20%) I’m always connected = 246 (18%)
Which device do you use most to surf the Internet?	Smartphone = 1234 (90%) Personal Computer/Laptop = 92 (7%) Tablet = 16 (1%)
What is your main reason for using Internet? (more than one answer)	Social Network = 976 (72%) Listen to music = 696 (51%) School research = 415 (30%) Play videogame = 327 (24%)
Do you talk about personal things on the Social Media that you wouldn’t share with others in person?	Never = 998 (73%) Sometimes = 252 (18%) Often = 87 (6%)
Do you think it’s easier to be yourself on the Social Media than in person?	Never = 838 (61%) Sometimes = 370 (27%) Often = 134 (10%)
Can you talk about more things on Social Media than you do in person?	Never = 924 (68%) Sometimes = 300 (22%) Often = 117 (9%)
Do you ever find yourself staying up late on Social Media?	Sometimes = 578 (42%) Never = 353 (26%) Often = 260 (19%) Very Often =149 (11%)

### 3.3 Descriptive statistics of the main study variables

Descriptive statistics for the main study variables, including sample size, observed range, mean, and standard deviation, are reported in **Table 3**.

**Table 3:** Descriptive statistics of the main study variables.

Variable	N	Range	Mean	SD
Time spent on social media (Social Media Questionnaire)	1351	0–4	2.37	1.02
Private online self-disclosure (Social Media Questionnaire)	1332	0–6	1.19	1.40
Depressive Symptoms (Children Depression Inventory 2 – CDI-2)	1364	0–51	11.84	8.01
Somatic complaints Youth Self-Report – YSR/11-18	1364	0–10	1.99	1.98
Suicidal ideation Youth Self-Report – YSR/11-18	1312	0–2	0.13	0.44

SD , Standard Deviation.

### 3.4 Correlation analysis

Bivariate correlations among time spent on social media, private online self-disclosure, depressive symptoms, somatic complaints, and suicidal ideation are reported in **Table 4**.

**Table 4:** Bivariate correlations among the main study variables.

Variable	1	2	3	4	5
1. Time spent on social media	—				
2. Private online self-disclosure	.144**	—			
3. Somatic complaints	.138**	.169**	—		
4. Suicidal ideation	.068*	.236**	.316**	—	
5. Depressive Symptoms	.252**	.336**	.439**	.386**	—

Values are Pearson correlation coefficients. Pairwise Ns ranged from 1, 288 to 1, 364. p <.05; p <.01, two-tailed.

### 3.5 Hierarchical regression analysis

A hierarchical linear regression analysis was conducted to examine the incremental association of digital engagement variables, depressive symptoms, and somatic complaints with suicidal ideation (**Table 5**, **Table 6**). Age and gender were entered in the first block, time spent on social media and private online self-disclosure in the second block, depressive symptoms measured using the CDI-2 Total Score in the third block, and somatic complaints in the final block. The regression analysis was conducted on participants with complete data for all variables included in the model, resulting in a final analytic sample of 1, 160 adolescents. Linear regression diagnostics confirmed that model assumptions were met: visual inspection of P-P plots and residual scatterplots demonstrated acceptable normality and homoscedasticity, while multicollinearity diagnostics indicated no relevant collinearity among predictors, with tolerance values ranging from.660 to.883 and VIF values ranging from 1.132 to 1.516.

**Table 5:** Hierarchical linear regression predicting suicidal ideation.

Model	Predictor	B	SE	95% CI	β	t	p	Tolerance	VIF
Model 1	Age	0.018	0.006	[0.007, 0.030]	.092	3.14	.002	.999	1.001
	Gender	-0.058	0.025	[-0.108, -0.009]	-.068	-2.33	.020	.999	1.001
Model 2	Age	0.018	0.006	[0.007, 0.030]	.092	3.09	.002	.908	1.102
	Gender	-0.067	0.025	[-0.117, -0.017]	-.078	-2.63	.009	.931	1.075
	Time spent on social media	-0.008	0.013	[-0.034, 0.018]	-.018	-0.57	.570	.832	1.202
	Private online self-disclosure	0.070	0.009	[0.052, 0.088]	.223	7.74	<.001	.976	1.025
Model 3	Age	0.008	0.006	[-0.003, 0.019]	.041	1.41	.159	.884	1.132
	Gender	-0.006	0.025	[-0.054, -0.043]	-.007	-0.24	.814	.884	1.131
	Time spent on social media	-0.025	0.013	[-0.050, -0.000]	-.060	-2.00	.046	.818	1.222
	Private online self-disclosure	0.036	0.009	[0.018, 0.054]	.114	3.91	<.001	.863	1.159
	Depressive symptoms (CDI-2)	0.019	0.002	[0.016, 0.022]	.339	11.00	<.001	.776	1.288
Model 4	Age	0.009	0.006	[-0.002, 0.020]	.044	1.55	.122	.883	1.132
	Gender	-0.020	0.025	[-0.029, 0.069]	.023	0.81	.420	.852	1.174
	Time spent on social media	-0.024	0.013	[-0.048, 0.001]	-.056	-1.90	.058	.818	1.223
	Private online self-disclosure	0.034	0.009	[0.016, 0.051]	.107	3.72	<.001	.862	1.161
	Depressive symptoms (CDI-2)	0.015	0.002	[0.011, 0.019]	.269	8.15	<.001	.660	1.516
	Somatic complaints (YSR)	0.037	0.007	[0.024, 0.050]	.168	5.46	<.001	.757	1.321

N = 1, 160. B = unstandardized coefficient; SE, standard error; β, standardized beta coefficient. Gender was coded as 0 = male and 1 = female.

**Table 6:** Goodness-of-fit parameters and incremental variance (Δ R²) across hierarchical regression blocks predicting suicidal ideation (N = 1, 160).

Model	R2	Adjusted R2	ΔR2	F	p	F-change	p-change
Model 1	.013	.012	.013	7.92	<.001	7.92	<.001
Model 2	.062	.059	.049	19.24	<.001	30.16	<.001
Model 3	.151	.148	.089	41.19	<.001	121.02	<.001
Model 4	.173	.169	.021	40.16	<.001	29.85	<.001

Model 1 included age and gender. Model 2 added time spent on social media and private online self-disclosure. Model 3 added depressive symptoms measured with the CDI-2 Total Score. Model 4 added somatic complaints.

All regression models were statistically significant. Model 1, including age and gender, explained 1.3% of the variance in suicidal ideation, R² = .013, adjusted R² = .012, F(2, 1157) = 7.92, p <.001. The addition of time spent on social media and private online self-disclosure in Model 2 significantly increased the explained variance, ΔR² = .049, F-change = 30.16, p <.001. Model 2 explained 6.2% of the variance in suicidal ideation, R² = .062, adjusted R² = .059, F(4, 1155) = 19.24, p <.001. The addition of depressive symptoms in Model 3 further improved the model, ΔR² = .089, F-change = 121.02, p <.001, with the model explaining 15.1% of the variance, R² = .151, adjusted R² = .148, F(5, 1154) = 41.19, p <.001. Finally, adding somatic complaints in Model 4 produced a further significant increase in explained variance, ΔR² = .021, F-change = 29.85, p <.001. The final model explained 17.3% of the variance in suicidal ideation, R² = .173, adjusted R² = .169, F(6, 1153) = 40.16, p <.001.

In the final model, private online self-disclosure, depressive symptoms, and somatic complaints were independently associated with suicidal ideation. Specifically, higher private online self-disclosure (B = 0.034, SE = 0.009, 95% CI [0.016, 0.051], β = .107, p <.001), higher depressive symptoms (B = 0.015, SE = 0.002, 95% CI [0.011, 0.019], β = .269, p <.001), and higher somatic complaints (B = 0.037, SE = 0.007, 95% CI [0.024, 0.050], β = .168, p <.001) were associated with greater suicidal ideation. Age, gender, and time spent on social media were not significant predictors in the final model.

As a sensitivity analysis, the final model was repeated using ordinal logistic regression, treating suicidal ideation as an ordinal outcome. The proportional odds assumption was supported by a non-significant test of parallel lines. The pattern of findings was consistent with the hierarchical linear regression: private online self-disclosure, depressive symptoms, and somatic complaints remained significantly associated with higher suicidal ideation, whereas age, gender, and time spent on social media were not significant predictors (detailed in Section 3.6 and **Table 7**).

**Table 7:** Ordinal logistic regression model predicting suicidal ideation (YSR item).

Predictor	B	SE	z	p-value	Odds Ratio (OR)	95% CI
Age	0.095	0.059	1.61	.108	1.10	[0.98, 1.23]
Gender (Male vs. Female)	-0.076	0.265	-0.29	.775	0.93	[0.55, 1.56]
Time spent on social media	-0.067	0.120	-0.56	.581	0.94	[0.74, 1.19]
Private online self-disclosure	0.253	0.075	3.37	.001	1.29	[1.11, 1.49]
Depressive symptoms (CDI-2)	0.099	0.016	6.19	<.001	1.10	[1.07, 1.14]
Somatic complaints (YSR)	0.229	0.054	4.24	<.001	1.26	[1.13, 1.40]

N = 1, 160. OR, Odds Ratio; CI, Confidence Interval. Overall model fit: Log-Likelihood = -328.21, χ² (6) = 241.15, p <.001. Test of parallel lines: p = .308.

To evaluate whether treating social media time as a continuous score affected the findings, the final regression model was re-estimated treating social media exposure as a categorical variable (dummy-coded across the 5 response categories). In the categorical model, none of the social media time categories were significantly associated with suicidal ideation (p-values ranging from.368 to.997), whereas private online self-disclosure (p <.001), depressive symptoms (p <.001), and somatic complaints (p <.001) retained their strong, independent statistical significance.

To address potential item overlap between the depressive symptom predictor and the suicidal ideation outcome, sensitivity analyses were performed using the refined CDI-2 score (excluding Item 8). In the refined multivariable model (N = 1, 160), refined depressive symptoms remained a strong independent predictor of suicidal ideation (B = 0.014, SE = 0.002, t = 7.55, p <.001). Private online self-disclosure (B = 0.035, SE = 0.009, t = 3.85, p <.001) and somatic complaints (B = 0.038, SE = 0.007, t = 5.69, p <.001) maintained their strong, independent associations, with somatic complaints accounting for a statistically significant additional 2.3% of unique variance (Δ R² = .023, p <.001). Time spent on social media remained non-significant (B = -0.023, p = .066). Parallel results were confirmed in the secondary ordinal logistic regression model using the refined CDI-2 score, where private online self-disclosure (OR = 1.29, 95% CI [1.11, 1.49], p = .001), somatic complaints (OR = 1.26, 95% CI [1.13, 1.40], p <.001), and refined depressive symptoms (OR = 1.10, 95% CI [1.07, 1.14], p <.001) independently predicted higher odds of suicidal ideation.

Restricting the analytic sample strictly to participants within the standardized normative age range of the tools (11–17 years; N = 978) fully replicated the primary findings. Private online self-disclosure (B = 0.028, SE = 0.010, t = 2.94, p = .003), depressive symptoms (B = 0.014, SE = 0.002, t = 7.22, p <.001), and somatic complaints (B = 0.037, SE = 0.007, t = 5.20, p <.001) remained independently and significantly associated with higher suicidal ideation, whereas time spent on social media remained non-significant (B = -0.016, SE = 0.014, t = -1.15, p = .252).

### 3.6 Ordinal logistic regression analysis

To complement the hierarchical linear regression and accommodate the ordinal distribution of suicidal ideation, an ordinal logistic regression model was estimated on the analytic sample (N = 1, 160). The test of parallel lines supported the proportional odds assumption (χ² = 7.14, p = .308), confirming the appropriateness of the model.

The pattern of results from the ordinal logistic regression fully replicated the primary linear regression findings. Greater private online self-disclosure was associated with a 29% increase in the odds of reporting higher suicidal ideation (OR = 1.29, 95% CI [1.11, 1.49], p = .001). Similarly, higher somatic complaints (OR = 1.26, 95% CI [1.13, 1.40], p <.001) and higher depressive symptoms (OR = 1.10, 95% CI [1.07, 1.14], p <.001) independently increased the odds of greater suicidal ideation. In contrast, time spent on social media (OR = 0.94, 95% CI [0.74, 1.19], p = .581), age (OR = 1.10, p = .108), and gender (OR = 0.93, p = .775) showed no statistically significant independent association with suicidal ideation (**Table 7**).

Identical patterns were observed when estimating the ordinal logistic model with the refined CDI-2 score (OR = 1.29, p = .001 for online self-disclosure; OR = 1.26, p <.001 for somatic complaints; OR = 1.10, p <.001 for refined depression) and across the 11–17 normative age subset (N = 978; OR = 1.24, p = .010 for online self-disclosure; OR = 1.27, p <.001 for somatic complaints; OR = 1.11, p <.001 for depression).

Given the modest internal consistency of the three-item private online self-disclosure measure, an additional sensitivity analysis examined its component items separately. The final multivariable model was re-estimated three times, replacing the composite score with each individual item while retaining the same covariates and analytic sample (N = 1, 160). Talking about private matters online that would not be shared in person showed the strongest independent association with suicidal ideation (B = 0.087, SE = 0.021, β = .118, p <.001, 95% CI [0.047, 0.128]). Perceiving it as easier to be oneself online than in person was also independently associated with suicidal ideation, although more weakly (B = 0.039, SE = 0.018, β = .061, p = .030, 95% CI [0.004, 0.074]). In contrast, reporting being able to talk about more things online than in person was not independently associated with suicidal ideation after adjustment for the remaining predictors (B = 0.031, SE = 0.019, β = .046, p = .104, 95% CI [-0.006, 0.068]). Parallel ordinal logistic regression analyses yielded the same pattern (OR = 1.84, p <.001; OR = 1.39, p = .033; and OR = 1.25, p = .167, respectively). These findings indicate that the association observed for the composite score was primarily supported by the behavioral disclosure component rather than uniformly by all three items.

## 4 Discussion

The present study examined the associations between social media use patterns, private online self-disclosure, depressive symptoms, somatic complaints, and suicidal ideation in a large school-based sample of Italian adolescents. Overall, the findings supported the study hypotheses. Higher levels of private online self-disclosure, depressive symptoms, and somatic complaints were associated with greater suicidal ideation. Importantly, private online self-disclosure showed a stronger association with suicidal ideation than time spent on social media, and remained significant even after controlling for depressive symptoms and somatic complaints. Depressive symptoms explained a substantial proportion of additional variance in suicidal ideation, while somatic complaints further contributed to the model beyond depressive symptoms and social media-related variables. These findings suggest that suicidal ideation in adolescence may be better understood by considering not only the amount of time spent online, but also the psychological meaning and function of online behaviors.

A primary clinical implication of our findings concerns the non-significant role of time spent on social media in the final model. This result directly challenges the simplified paradigm often in which the screen time alone is assumed to drive adolescent psychopathology (12, 20). In our sample of adolescents, suicidal ideation was independently associated with depressive symptoms, somatic complaints, and qualitative aspects of digital engagement, particularly private online self-disclosure, rather than with time spent on social media alone. This result is consistent with an increasing body of literature suggesting that screen time alone may be an insufficient indicator of psychological risk in adolescents (12, 15, 20). Several studies and reviews have emphasized that the relationship between digital media use and adolescent mental health is complex, heterogeneous, and likely influenced by individual vulnerability, social context, type of online activity, and subjective meaning of use (5, 8, 13, 20). In this perspective, the present findings support the need to move beyond purely quantitative measures of digital exposure and to focus on qualitative dimensions of online engagement.

In line with this interpretation, private online self-disclosure emerged as an independent correlate of suicidal ideation. At the composite level, greater private online self-disclosure was independently associated with higher suicidal ideation even after accounting for depressive symptoms and somatic complaints. However, the item-level sensitivity analyses provided a more nuanced picture. The association was strongest for disclosing private matters online that would not be shared in person and remained significant, although weaker, for perceiving it as easier to be oneself online than in person, whereas the broader tendency to talk about more things online than offline was not independently associated with suicidal ideation after adjustment for the other predictors. These findings suggest that the observed association should not be generalized to online self-disclosure as a whole, but may be particularly relevant to forms of online expression characterized by a discrepancy between what adolescents disclose online and what they communicate in face-to-face contexts. According to the compensatory Internet use framework, online environments may provide opportunities to express experiences that are difficult to communicate offline or to seek connection and emotional support (14, 21). However, given the cross-sectional design, the present findings cannot establish whether these patterns of online disclosure contribute to psychological vulnerability, emerge in response to existing distress, or represent attempts to cope with unmet emotional or relational needs (14, 17).

Depressive symptoms represented the strongest predictor of suicidal ideation in the final model, confirming a well-established association between depression and suicidality in adolescence (22–25). This finding is consistent with epidemiological evidence showing that depressive symptoms are highly prevalent among adolescents and represent a major contributor to psychological burden and suicide risk (2, 26, 27). It also aligns with studies linking adolescent social media use, depressive symptoms, and suicidal ideation, while emphasizing that the direction and mechanisms of these associations remain debated (1, 5, 7, 9). Crucially, the present results do not support a causal interpretation whereby social media use directly produces suicidal ideation. Rather, they suggest that depressive symptoms are central to suicidal ideation and may partly account for the association between digital behaviors and mental health outcomes.

A particularly important contribution of this study is the observation that somatic complaints accounted for additional variance in suicidal ideation beyond depressive symptoms, time spent on social media, and private online self-disclosure. Although the increase in explained variance was modest, somatic complaints remained independently associated with suicidal ideation in the final model.

This finding suggests that somatic complaints may reflect an additional dimension of psychological distress not fully captured by depressive symptoms scales alone. In clinical and developmental literature, emotional distress in adolescents is frequently expressed through physical symptoms such as headache, nausea, dizziness, or other physical complaints. Within school and primary care settings, somatic complaints may represent an observable correlate of psychological distress, although the present cross-sectional findings cannot establish their temporal or predictive value in relation to suicidal ideation.

Crucially, time spent on social media lost its residual significance across all multivariable regression models once qualitative online engagement, depressive symptoms, and somatic complaints were included into the model. The incremental role of somatic complaints carries relevant clinical implications. Adolescents reporting recurrent somatic complaints, particularly in combination with depressive symptoms or private online self-disclosure, may represent a subgroup characterized by greater psychological vulnerability and therefore deserving further investigation in prospective studies. Somatic complaints should therefore not be interpreted only as isolated physical symptoms, but also as possible indicators of emotional dysregulation, internalizing distress, or difficulty directly verbalizing psychological suffering. This is consistent with broader clinical literature emphasizing the overlap among physical symptoms, depressive manifestations, and emotional vulnerability in adolescent populations (28). The present findings indicate that somatic complaints account for additional variance in suicidal ideation beyond depressive symptoms, although their potential clinical utility requires further investigation in prospective studies.

To contextualize why somatic complaints provided incremental value in predicting psychological vulnerability, the potential link between digital engagement habits and physical symptoms warrant theoretical consideration. In our descriptive data, a notable proportion of adolescents reported engaging with social media late at night. Extant literature suggests that nocturnal digital media use may involve both physiological influences—such as screen-light exposure potentially interfering with circadian sleep regulation (29, 30)—and psychological influences, including emotional overstimulation from peer interactions prior to sleep. In broader clinical models, chronic sleep disruption and associated somatic distress frequently present as unexplained physical symptoms—primarily headaches, dizziness, and nausea (31, 32)—which constitute core items of the somatic complaints index evaluated in this study. However, because sleep quality, melatonin levels, and autonomic markers were not directly measured in our study protocol, these neurobiological pathways remain plausible theoretical hypotheses derived from external literature rather than directly observed mechanisms. Future prospective research incorporating objective sleep tracking and physiological assessments is required to empirically test whether sleep disruption mediates the association between late-night digital engagement and somatic complaints.

Within the developmental framework of adolescence, the body often serves as a key channel for expressing emotional distress, particularly when psychological discomfort remains unrecognized or difficult to verbalize. From a psychosomatic perspective, chronic somatic complaints may co-occur with internalizing distress; however, the present findings do not allow these symptoms to be attributed specifically to digital fatigue or to any particular underlying mechanism. Persistent somatic complaints may co-occur with heightened psychological vulnerability and internalizing distress during adolescence (33, 34). While longitudinal research is necessary to establish directional pathways, our findings suggest that somatic complaints may provide additional information about adolescents’ psychological vulnerability beyond depressive symptoms. Further prospective research is needed to determine whether these complaints have predictive or clinical utility in identifying adolescents experiencing greater psychological distress. The results also have implications for prevention and school-based mental health programs. Given the widespread use of social media in the sample, universal approaches based exclusively on reducing screen time may be insufficient. Instead, the present findings suggest that future research and prevention efforts may benefit from considering how adolescents use digital environments and the functions that online communication may serve. In particular, the context of online self-disclosure and its relationship with available offline support warrant further investigation before specific clinical or screening implications can be established. At the same time, digital spaces should not be framed only as harmful environments. For some adolescents, online communication may represent an accessible channel for self-expression or support. Future research should therefore examine the contexts and functions of online disclosure to determine when these behaviors may be associated with psychological distress or, conversely, may serve as forms of self-expression, connection, or support seeking.

Several limitations of the present study should be acknowledged. First, the cross-sectional design precludes any conclusions regarding causal or temporal relationships. It is not possible to determine whether private online self-disclosure, depressive symptoms, or somatic complaints contribute to suicidal ideation, or whether adolescents experiencing suicidal ideation are more likely to disclose private experiences online and report somatic complaints. Longitudinal prospective studies are needed to clarify directionality and developmental trajectories. Second, all variables were assessed through self-report measures, which may be susceptible to recall bias, social desirability, or underreporting, particularly for sensitive internalizing issue such as suicidal ideation. Third, suicidal ideation was assessed using a single YSR item. Although single-item screeners are clinically meaningful and widely used in epidemiological surveys, future studies should incorporate multidimensional assessment of suicidality, including frequency, intensity, explicit planning, intent, and non-suicidal self-injury. Fourth, private online self-disclosure was assessed using a brief, study-specific three-item composite with modest internal consistency (Cronbach’s α = .57) and without prior psychometric validation. Accordingly, the composite should be interpreted as a preliminary indicator of qualitative digital engagement rather than as a validated unitary psychological construct. Item-level sensitivity analyses further showed that the association with suicidal ideation was not uniform across all three components and was primarily supported by the item assessing disclosure of private matters online that would not be shared in person. This heterogeneity limits the precision and generalizability of conclusions concerning the broader construct of private online self-disclosure. Future research should replicate these findings using validated multidimensional instruments capable of distinguishing behavioral disclosure, online authenticity, disclosure breadth, disclosure context, and the intended audience or communication channel. Fifth, the study was conducted in secondary schools from a specific region of Southern Italy, which may limit generalizability to adolescents from distinct geographical, cultural, or socioeconomic contexts. Sixth, the analytic sample was restricted to participants with complete data across all study variables, though attrition analysis indicated no systematic bias regarding primary clinical variables. Finally, the multivariable model explained a meaningful but moderate proportion of variance in suicidal ideation (17.3%), indicating that unmeasured parameters—including family functioning, bullying and cyberbullying, objective sleep parameters, trauma exposure, loneliness, body image concerns, and clinical psychiatric history — should be included in future studies (35–44).

Despite these limitations, the present study contributes to the literature by highlighting the importance of qualitative dimensions of digital engagement and somatic complaints in relation to adolescent suicidal ideation. The findings suggest that private online self-disclosure may provide more meaningful insight into psychological vulnerability than quantitative time spent on social media. Moreover, somatic complaints accounted for incremental variance beyond depressive symptoms, although their potential screening or clinical utility requires prospective validation. Overall, these results underline the need for multidimensional approaches to adolescent mental health in the digital age, integrating qualitative digital behaviors, emotional symptoms, bodily complaints, and protective psychological resources.

## 5 Conclusion

In conclusion, the present study suggests that suicidal ideation in adolescence is associated not simply with the amount of time spent on social media, but with the way adolescents use online spaces to express private aspects of themselves. Private online self-disclosure, depressive symptoms, and somatic complaints were independently associated with suicidal ideation, whereas time spent on social media was not significant in the final model. These findings support the relevance of assessing qualitative online behaviors and somatic complaints as part of a broader evaluation of adolescent psychological vulnerability. For psychiatrists, psychologist, and other mental health professionals, these findings highlight the potential relevance of looking beyond screen duration to consider qualitative aspects of adolescents’ digital experiences. Future prospective studies should determine whether assessing the context and function of online communication, together with emotional and somatic symptoms, provides clinically useful information beyond measures of screen time alone. Longitudinal research is essential to clarify temporal and potentially causal pathways and to determine how depressive symptoms, somatic complaints, and different patterns of online self-disclosure relate to suicidal ideation over time.

## Data Availability

The datasets presented in this study can be found in online repositories. The names of the repository/repositories and accession number(s) can be found below: https://osf.io/n3d7e/overview?view_only=1e76c74aae62430d84d5f31fdd59c427.
